# Public views on research with publicly available data in Switzerland: Implications for digital research, science communication, and policy

**DOI:** 10.1177/09636625251330575

**Published:** 2025-05-22

**Authors:** Paola Daniore, Jana Sedlakova, Federica Zavattaro, Zoé Huber, Melanie Knieps, Manon Haulotte, Togbé Agbessi Alangue, Artemis Faulk, Viktor von Wyl, Yaniv Benhamou, Felix Gille

**Affiliations:** Swiss Federal Institute of Technology Lausanne (EPFL), Switzerland; University of Zurich, Switzerland; University of Geneva, Switzerland; University of Zurich, Switzerland; University of Fribourg, Switzerland; University of Geneva, Switzerland; University of Zurich, Switzerland; University of Geneva, Switzerland; University of Zurich, Switzerland

**Keywords:** artificial intelligence, digital research, publicly available data, qualitative research, Switzerland, trust

## Abstract

The increasing volume of publicly available data brought about by digitalization offers researchers opportunities to examine public sentiment on various national and global issues. However, concerns linked to the use of publicly available data in digital research are insufficiently addressed. To ensure its ethical and trustworthy conduct, it is crucial to assess the public’s perception of digital research with publicly available data. We conducted 10 focus groups with 75 participants from the German-, French-, and Italian-speaking regions in Switzerland, reflecting nationwide perspectives on digital research with publicly available data. Through a thematic analysis, four major themes emerged: (1) expectations toward actors and digital research with publicly available data, such as alignment with research standards to promote result validity, using research findings for societal benefit, and ensuring transparency on data use through informed consent; (2) concerns about data reuse for purposes beyond the study’s objectives, especially for financial gain, as well as concerns about method reliability, data quality, and privacy; (3) mitigative measures to minimize potential harm, such as through the involvement of external oversight committees; and (4) supportive measures encompassing communication strategies to raise awareness and inform the public about the use of their data for research purposes. Our findings suggest public support for digital research with publicly available data provided that specific expectations are met. Developing a framework for legitimate digital research with publicly available data is identified as a valuable next step, with a focus on broadening public awareness on digital research with publicly available data through nationwide communication campaigns and introducing relevant oversight measures to foster trust.

## 1. Introduction

The growing digitalization of society has led to the increased generation and accessibility of publicly available data. Publicly available data can be defined as data not generated for research purposes that can be found and accessed easily and often for free (Cooper and Coetzee, 2020; Stommel and Rijk, 2021). This includes a wide range of online data types, such as social media posts, newspaper articles, or real world data from open access repositories. Conducting academic research with publicly available data typically requires digital methods for their collection and analysis, such as applying natural language processing tools to assess public sentiment on current issues from large-scale text data. Consequently, in this study we refer to “digital research with publicly available data” as the use and analysis of publicly available data facilitated by digital methods for secondary research (Snee et al., 2016).

Publicly available data can be leveraged to enrich research studies by providing diverse datasets that enable deeper analysis and novel insights across all disciplines. For example, this data can be used in the field of social sciences to understand public sentiment on national and global issues, including climate change and public health measures, or to evaluate the impact of policy changes (Blasimme et al., 2018; Schneider et al., 2023). To ensure high research quality, uphold its benefits for society (Barrow et al., 2023) and maintain public trust in academic research (Gille et al., 2021; Kerasidou, 2017), scientific activity is grounded in research ethics. However, unlike primary research, individuals involved in secondary research are often unaware that their data is being used, which undermines the essential research ethics principles of informed consent, data privacy, and transparency (Bak et al., 2023; Ferretti et al., 2021). For example, citizens who share data on public platforms, such as X (formerly Twitter), may unknowingly contribute to research, as the terms and conditions governing data use are often lengthy, written in complex legal language, frequently ignored, and in general hard to navigate (Ravn et al., 2020; Stommel and Rijk, 2021). At the same time, there is a growing demand for transparency regarding how personal data is used in research (Anhalt-Depies et al., 2019; Murphy et al., 2021). This is coupled with increasing criticism of the insufficient engagement between academic and non-academic stakeholders in shaping research practices (Huzzard, 2021). Not addressing these concerns in digital research with publicly available data can further disconnect the research community from society, undermining public trust and participation in research.

### The challenge of legitimizing digital research with publicly available data

Research efforts so far have focused on exploring best practices to conduct research that addresses methodological and ethical challenges introduced by online data and digital methods (Gliniecka, 2023; Ravn et al., 2020; Stommel and Rijk, 2021). This includes the development of recommendations for ethical Internet research (Buck and Ralston, 2021; Stommel and Rijk, 2021; The Association of Internet Researchers, 2019), privacy and data protection (Blasimme et al., 2018; Vayena et al., 2018), and trust-based governance of research (Bak et al., 2023). A recent scoping review assessed public involvement and engagement strategies for supporting digital research, highlighting its potential to foster public trust and accountability in data reuse for research (Teodorowski et al., 2023). The review highlights the persistent lack of empirical studies on public involvement in big data research, hindering efforts to legitimize this type of research. A recent review of studies that use public data in their research found that one-third did not address ethical issues, often because the data was labeled as public and therefore assumed to not require special ethical considerations. When ethical concerns were mentioned, there was significant variation in how they were handled, with minimal discussion of user intentions, potential harm, or consent (Stommel and Rijk, 2021).

For digital research with publicly available data specifically, guidelines at the national and international level have been published to inform science communication efforts (National Institutes of Health (NIH), 2018; Open Research Europe, 2023; Swiss Academies of Arts and Sciences, 2021). Applying these principles to involve the public in digital research with publicly available data could be beneficial to democratize research, bringing the public closer to the research community and improving the efficacy of science communication efforts (Binder et al., 2012; de Sherbinin et al., 2021). However, a comprehensive investigation involving the public in defining the legitimacy of digital research with publicly available data is still lacking. Such involvements have the potential to enable more holistic and deeper ethical discussions to address the challenges of digital research (Stommel and Rijk, 2021).

To maintain trust in digital research, it is therefore essential to incorporate the public’s opinion. This study investigates the perceptions and attitudes of the Swiss public toward digital research with publicly available data, focusing on the conditions that foster their support and their concerns. The following study aims were defined to inform the data collection:

1. To identify the conditions for acceptance and support of digital research with publicly available data;
2. To determine whether public perceptions vary based on who is conducting this type of research, the context of the research, and the types of data involved;
3. To identify gaps and requirements in science communication on digital research with publicly available data research with the goal of fostering public support and trust.

The overarching objective of this study is to incorporate public perspectives on digital research with publicly available data into the development of research governance, science communication, and policymaking.

## 2. Methods

### Theoretical foundations

In our study we follow a grounded theory approach to assess public conditions for the acceptance and support of digital research with publicly available data in Switzerland. This approach involved iterative data collection and analysis, where initial focus group findings guided subsequent sessions and helped refine the questions and topics explored. By following a grounded theory approach, the study’s methods did not rely on guiding hypotheses or on any pre-existing theoretical framework. However, the preliminary theoretical understanding of ethical data use for this study is guided by discussions in Internet research ethics highlighting problems of informed consent, surveillance, anonymization, data interpretation, and privacy (Buck and Ralston, 2021; Catone, 2023; Gliniecka, 2023; Kalfoglou, 2016). Internet research ethics emphasize the need to balance respect for individual privacy with the public nature of online data. This discussion helps contextualize public concerns about digital research, particularly regarding how data that are publicly accessible can still involve ethical considerations such as problems of surveillance, the expectations of privacy by individuals, and the appropriate use of such data by researchers. By grounding the study in these ethical principles, this study explores public opinion with a more holistic understanding of the ethical implications and societal expectations surrounding digital research practices. These concepts, along with guiding principles for building trust in academic research (Resnik, 2011), form the theoretical foundation for the data collection process.

### Study sample and design

We conducted ten focus groups across the German-, French-, and Italian-speaking cantons of Switzerland to capture nationwide public perspectives on digital research with publicly available data. For the purposes of this research study, “the public” refers to a group of participants who are reflective of the Swiss population, with no prior knowledge or specific expertise in digital research with publicly available data. This group is intended to provide a general perspective on digital research with publicly available data from individuals who are typical members of the community. Among the focus groups, four were conducted with participants from the German region, four with participants from the French region, and the remaining two with participants from the Italian region. All focus groups were conducted in the language of the respective region, namely in German, French, and Italian. Recruitment, screening, and informed consent for the focus groups were undertaken by gfs.bern, a market research agency (gfs.bern—Political and communications research, 2019). Participants were carefully selected for the focus groups to ensure a balanced representation of different age groups and political preferences, with the aim to reflect the diverse views of the Swiss public. The study design was reviewed by the Zurich cantonal ethics commission (BASEC-NR. 2022-00580), the Geneva cantonal ethics commission (BASEC-NR. 2023-00212), and University of Geneva ethics commission (CUREG-2023-03-41).

The focus groups were conducted on Zoom’s video conferencing platform (version 5.7.7) and followed a semi-structured format guided by two approaches. First, we presented participants in each language group different case studies derived from real-world examples. The topic areas of the case studies were chosen based on the experiences of the research team and that encompass a diverse range of research topics and digital methods with publicly available data. The following case studies were presented to the participants: (1) using Twitter data to assess public opinion on gene-editing technology, (2) assessment of data sharing policy documents, (3) using social media data to predict an individual’s health status, and (4) using YouTube comment data to assess public sentiment on health policy developments (Blasimme et al., 2018; Coppersmith et al., 2018; Müller et al., 2020; Porreca et al., 2020). These case studies showcased how digital research with publicly available data can be applied. Second, we guided discussions by focusing on key categories related to digital research with publicly available data (Schneider et al., 2023), such as relevant actors and the types of data used in the studies. We chose to guide the discussions with pre-defined categories to assist participants in navigating the complexities of digital research with publicly available data, allowing for more meaningful and structured discussions (Bowling and Ebrahim, 2005).

### Data collection and analysis

**Figure 1.**
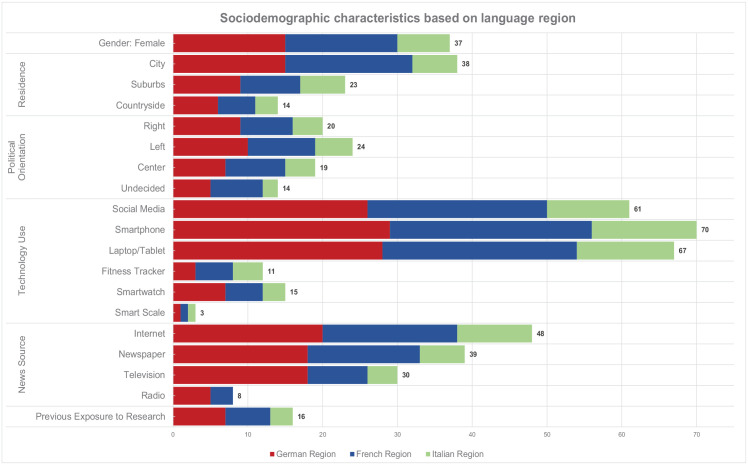
Baseline demographics.

**Figure 2.**
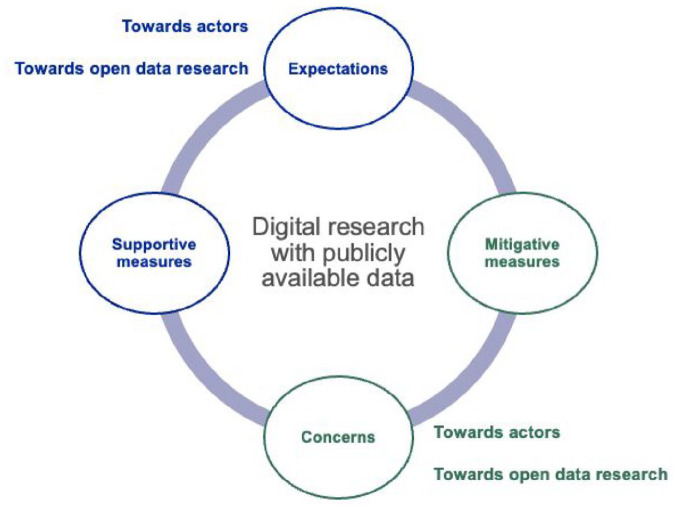
Overview of themes and subthemes.

Data was collected between September 2022 and June 2023. Each focus group interview was conducted by a team of researchers taking on various roles, including observation, note-taking, and moderation. Before the start of the focus groups, participants were asked to complete an online questionnaire (Qualtrics, version 12018) to collect baseline sociodemographic data, technology use and affinity, as well as understanding of digital research with publicly available data (Figure 1). Written data from the focus groups was collected using an online whiteboard software (Flinga, version 6) and structured in real-time based on the pre-defined categories. Participants had the opportunity to contribute to the data collection by adding notes themselves to the online whiteboard. No audio or video recordings of the focus groups were taken. The data was then translated to English using DeepL (deepl.com, version 1.17.1 to 2.4.0) and imported into MaxQDA (version 10), a qualitative data analysis software (VERBI Software, 2021). Translation was examined for accuracy by bilingual research team members. The data was then coded and analyzed following an inductive thematic approach, which was conducted over two phases (Pope et al., 2020). In the first phase, the data was kept in the original categories used for the data collection and coded inductively by one author (PD), followed by iterative conceptualization cycles for theme development with other authors (FG, JS). The codes were mapped with specific subthemes determined by thematic similarities within each category. Similar subthemes across all categories were then grouped based on their commonalities (Supplemental Table 1). In the second phase, the grouping of the subthemes was informed by the study’s guiding questions to provide concrete insights for future guidance development (Figure 2).

This study was a collaborative effort between the University of Zurich and the University of Geneva. In addition to the core research team involved in the study, part of this study was conducted in collaboration with students participating in the Digital Clinics course at the University of Geneva. Interviews for the German and Italian region focus groups were conducted by the core research team (FG, PD, JS, FZ). Interviews for the French region focus groups were conducted by students from the University of Geneva and a core member of the research team (PD). All focus groups for each language region reached thematic saturation, which was identified by the completeness and replicative nature of the data from the focus groups from each language region.

## 3. Results

### Participant demographics

The participants’ baseline data is summarized in Figure 1. Overall, a total of 75 participants took part in the focus groups. Approximately half of the participants were female (n = 37, 49%), most participants lived in a city (n = 38, 51%) and were social media users (n = 61, 84%). Prior to the conduct of this study, only few participants (n = 16, 22%) had heard about digital research with publicly available data.

### Themes

We identified four major themes describing the Swiss public’s perspectives on digital research with publicly available data: (1) Expectations; (2) Concerns; (3) Mitigative measures; and (4) Supportive measures (Figure 2). Sub-themes were identified within the major themes to highlight key distinctions observed in the data. While these themes are not directly aligned with the study’s research aims, they provide a comprehensive overview of public opinions that is both data-driven and descriptive.

The themes of Expectations (1) and Mitigative measures (3) are particularly relevant to our first research aim, which seeks to examine the conditions for acceptance and support of digital research with publicly available data. The sub-themes identified under Expectations (1) and Concerns (2) are particularly important for addressing the second research aim, which explores differences in public opinion depending on who is conducting the type of research, the context of the research, and the types of data involved. Finally, the theme of Supportive measures (4) is central to our third research aim, which investigates gaps and needs in science communication regarding digital research with publicly available data.

The first theme “Expectations” contains topics related to participants’ values and beliefs about relevant factors that will bring positive outcomes from the digital research with publicly available data. The second theme “Concerns” summarizes expressed challenges related to digital research with publicly available data. The third theme “Mitigative measures” includes specific actions to prevent and minimize potential negative impacts related to digital research with publicly available data on public trust (Table 1). The fourth and final theme “Supportive measures” includes provisions to assist or improve digital research with publicly available data in a way that is deemed legimate by the public.

**Table 1.** Summary of main themes and subthemes.

Theme	Subtheme/Main points	Description
Expectations	Of actors: - Good cause - Benefit to society - Awareness - Public information - Transparency - Agency About digital research with publicly available data: - High research standards - Relevance of results - Precautions for sensitive topics	*Public expectations are placed on researchers conducting digital research with publicly available data that adheres to high research standards and is beneficial to society, while also actively raising public awareness about digital research with publicly available data.*
Concerns	About actors: - Conduct research for other purposes - Implications of research findings - Intentions of private actors vs public actors - Lack of neutrality and objectivity About digital research with publicly available data: - Quality of data - Validity of methods - Protection of vulnerable groups - Reliability of AI algorithms - Data protection	*Public concerns regarding digital research with publicly available data center around the potential misuse of their data for purposes other than the intended study, questioning the validity and objectivity of research conducted with publicly available data, and the commercialization of their data by private actors.*
Mitigative measures	- External oversight - Researcher accountability - Guiding framework - State and global regulation - Right to be forgotten - Opt-in vs opt-out	*To mitigate public concerns of digital research with publicly available data, suggested measures include instating external oversight for digital research with publicly available data studies to increase researcher accountability, the development of a guiding framework to inform the trustworthy conduct of digital research with publicly available data, and updated opt-in or opt-out models to give the public more agency on their involvement in digital research with publicly available data.*
Supportive measures	- Public communication - Individual communication - Informed consent - Dynamic consent	*To support public expectations of digital research with publicly available data, suggested measures include public communication campaigns on digital research with publicly available data, individual notifications on the use of one’s publicly available data in a research study, and updated informed consent models.*

#### Theme 1: Expectations

##### Subtheme 1a: Expectations of actors

Participants discussed expectations that were related to different actors, such as researchers, companies, and the individuals themselves. Participants distinguished between public and private actors when discussing motivations for conducting digital research with publicly available data. Some participants expect that public actors would use publicly available data in research for a good cause, while they believe that private actors are mainly driven by profit. Regardless of whether the actors behind a study are public or private, there is the expectation that the actors align their studies with the public’s interest and use the findings to benefit the public and society at large, such as through policy implementations.

Another reoccurring theme was awareness. Awareness was raised in a rather general context of knowing that the data is used for different purposes and being aware of the risks when sharing information online. There was a widespread expectation of being sufficiently informed about the use of publicly available data for digital research. There were two opposite opinions about how to achieve this goal. Some participants advocated for relying on personal agency and self-responsibility to ensure they are well-informed, suggesting that individuals should take the initiative to read through the terms and conditions of the data platform and decide for themselves whether their data should be used. Other participants expected more transparency, including how the data is used, by whom and for what purposes. Two approaches to achieve this transparency stood out: participants expected to be given either (1) *more* information on the use of their data or (2) information of *higher quality*. Overall, participants placed importance on having access to information and the ability to make educated judgments on the use of publicly available data in digital research in the context of their personal agency and control over one’s data.

Participants who expected additional information about the use of their data for digital reach expressed the importance of appropriate informed consent procedures, regardless of whether the research studies were conducted by public or private actors. Informed consent forms were expected to provide, at the very least, clear specifications of the study’s objectives, duration, and approaches to data collection and sharing. Along these lines, participants also suggested the provision of new informed consent forms in case of changes to the study objectives or design. Participants who expected better quality information also called for clearer formulation of terms and conditions when they access online platforms, as well as more user-friendly approaches to define concepts that are not easily understood by laypeople within them. Other participants expected a precisely formulated informed consent form that would be limited to a specific time period and study objective. The participant discussions implied that the fulfillment of these expectations might lead to increased trust toward actors using publicly available data for digital research.

##### Subtheme 1b: Expectations specific to digital research with publicly available data

Particularly when discussing digital research with publicly available data, participants expected that the data is used as a means to assess public opinion on topics of public interest, as well as to answer questions that are of societal relevance. Participants expected that digital research methods are chosen to align with research standards and are appropriately implemented to ensure the validity of results for the populations of interest to the study. For some participants, it was important that researchers limit the use of publicly available data to cases where no other dataset is available. When discussing different case studies, participants’ expectations varied based on their perceived personal relevance of the study. For example, when discussing a case study on using publicly available policy papers in a research study, participants had minimal expectations regarding the study’s scope and the approaches researchers use to collect and analyze data. In contrast, when presented with a case study with the aim to predict health status using publicly available data, participants had high expectations due to the sensitive nature of the research, advocating for additional precautions. Overall, participants found that an appropriate study design, alignment with research standards, and relevance to the public are crucial factors for supporting and accepting the use of publicly available data for research.

#### Theme 2: Concerns

##### Subtheme 2a: Concerns about actors

Across all focus groups, concerns were raised about the use of publicly available data by actors for purposes beyond a study’s objectives. Some participants expressed concerns that individuals accessing their publicly available data may not have the intention of conducting research altogether but might have other motives or objectives for collecting and analyzing such data. For instance, private actors may collect publicly available data to inform business decisions with the ultimate goal of generating more profit. This concern became more pronounced when participants discussed how private actors could use publicly available data to draw conclusions on sensitive topics, such as the health status of a population, raising fears that it could be exploited for commercial gain by pharmaceutical or insurance companies, at the expense of the public. Participants also voiced concerns about public actors, such as universities or government entities, potentially using publicly available data for research studies not to address certain societal questions as claimed, but rather to advance their own agendas, such as pursuing policy or career goals. In turn, participants raised concerns about researchers’ ability to maintain neutrality and objectivity while using publicly accessible data for digital research.

##### Subtheme 2b: Concerns about digital research with publicly available data

Participants raised concerns about the quality of publicly available data, with specific concerns placed around bias and representativeness of the data. In particular, participants expressed that these issues may limit conclusions of the studies to specific sociodemographic groups, political perspectives, or members of the public who are more inclined to share data on the public domain. Going deeper into types of data suitable for digital research with publicly available data, participants raised concerns on the use of data that may reveal sensitive information about an individual if linked to other data sources, such as data related to financial or health status. Participants generally approved of using publicly available text data for digital research, but had reservations about picture, video, or audio data due to this data’s perceived sensitivity. Participants also had a negative perception of the use of publicly available data belonging to vulnerable groups, such as children.

Discussions in the focus groups also revolved around the reliability of methods employed in digital research with publicly available data. Exploring methods used for digital research with publicly available data, participants raised concerns about the reliability of AI-based algorithms and the accuracy of the data analysis when such algorithms are used. However, participants also expressed that their lack of understanding regarding the methods used in digital research with publicly available data prevents them from having an informed opinion in support of such research or against. Overall, participants agreed that methods relying on algorithms with limited or no researcher involvement were a source of concern. Participants particularly raised concerns around data protection and the ability of research methods to ensure that their data is secure, anonymized, and that their privacy preserved. Participants expressed doubts about the feasibility of data protection through anonymization, citing their lack of understanding of the process as a factor diminishing their confidence in its ability to protect their data from re-identification. Participants particularly raised concerns about making health-related data available for research purposes altogether.

#### Theme 3: Mitigative measures

Participants emphasized the role of external actors in setting appropriate research standards, precautions, and governance frameworks for digital research with publicly available data. Generally, participants found that ethics committees play an important role in ensuring that proper research standards are set in place. However, most participants called for the involvement of external oversight committees to further enforce the appropriate use of their data in digital research. For example, they suggested that external oversight committees include advisory commissions, controllers, and data protection officers that are independent of the research studies’ funding. Despite limited awareness of specific requirements or existing guidelines for the appropiate use of certain types of publicly available data in research, participants stressed their significance and highlighted the important role of external committees in enforcing them. Other participants placed the topic of responsibility and consequently accountability on the side of researchers. This was particularly the case in terms of alignment with ethical standards and best practices in research. There were also participants suggesting that there might be a need for a third-party body to control whether the purpose, methods, and design of studies are appropriate and that transparency criteria are met.

Overall, most of the discussions on mitigative measures emphasized the need for a framework to guide digital research with publicly available data and appropriate measures for collecting, analyzing, and reusing publicly available data. Throughout the focus groups, the significance of the state and government’s roles in ensuring researchers’ appropriate conduct in digital research with publicly available data was also highlighted. From a legislative standpoint, some participants believed that regulation for the use of publicly accessible data is necessary not only at the state level but also on a global scale due to data crossing borders, with emphasis on the importance to protect minors. Participants also discussed the “right to be forgotten,” expressing support for legislation but also questioning its practical feasibility. Finally, participants emphasized the importance of implementing consequences in cases of non-compliance with legislation or frameworks. When deciding whether to participate in a study, some participants preferred the option to actively opt into a study, while others were comfortable with a broader opt-out approach. In the latter case, some participants stated they would be comfortable with specifying the general conditions under which they would accept their publicly available data to be used in research, which would allow for the use of their data in various studies. By comparison, the participants who preferred an opt-in process believed it was necessary for studies on sensitive topics, such as those related to health status or finance.

#### Theme 4: Supportive measures

The most pronounced supportive measure expressed by participants was the use of communication strategies to increase public awareness on digital research with publicly available data. For instance, participants proposed leveraging science communication approaches, public campaigns to increase awareness on digital research with publicly available data, as well as prevention initiatives to educate minors about sharing data in the public domain, for example in schools. However, participants had varying views on who would be an appropriate communicator for these strategies. Some participants preferred public actors such as journalists, while others preferred researchers for this role, highlighting that researchers should be educated in appropriate science communication approaches for laypeople. Participants also called for regular communication on digital research with publicly available data developments across different platforms, including the radio, newspaper, television, and on the Internet. This could take the form of commercials on YouTube or in news segments to raise awareness on the reuse of data shared in the public domain, or scientists communicating interesting research findings, as well as their implications for the public.

Establishing a communication channel with individuals engaged in digital research with publicly available data may support their involvement in research. Foremost, participants suggested that they would prefer to be notified if their data may be used in a study in the first place. Upon learning that their data could be used for a study, participants suggested informed consent forms clearly stating the study’s objectives, duration, and approaches to data collection and sharing could be beneficial to make a more informed decision on whether they want to take part in a study. Finally, participants found that the communication between researchers and individuals whose publicly available data is used for the studies should be regular, and that relevant changes, such as in the terms and conditions, as well as study results, be communicated to them.

## 4. Discussion

### Main results

We identified four themes relevant to the Swiss public to support and accept digital research with publicly available data: (1) Expectations, (2) Concerns, (3) Mitigative measures, and (4) Supportive measures. These themes reveal the breadth and interrelated aspects associated with the public’s requirements for the involved actors, methods, analyses, and use cases when conducting digital research with publicly available data. To our knowledge, this is the first study to comprehensively assess public perspectives regarding digital research with publicly available data. The findings from this study can inform existing guidelines for fostering public involvement in digital research with publicly available data and support more targeted public communication efforts. In addition, they refine the broader understanding of public participation and trust in digital research by concentrating specifically on digital research with publicly available data. Furthermore, by focusing on a broad sample of the Swiss public across all age groups, levels of digital literacy, and geographical locations, this study captures a culturally diverse perspective, making its findings relevant and applicable to other international contexts.

### Facilitating conditions for public support of digital research with publicly available data

Our findings reveal that the public supports the use of publicly available data for digital research. Nevertheless, public support is conditional on how well researchers meet certain expectations. Generally speaking, a key expectation for public support relies on research that uses publicly available data to address topics that are of interest to and beneficial for society. This finding is consistent with efforts on behalf of researchers to inform the study design and disseminate research findings with a focus on societal benefits (Lawrence, 2006; Roberts, 2009). These efforts, however, are ineffective if the public is unaware of the conduct of this type of research in the first place. There was a consensus that the public should, at a minimum, be informed that their data may be used for a research study. Strategies in the form of funding schemes (Swiss National Science Foundation (SNSF), 2023), science communication guidelines (Swiss Academies of Arts and Sciences, 2021), and science journalism (SRF, 2020) exist in Switzerland. However, our findings echo the need for novel approaches to engage the public through awareness and empowerment over their own data’s vital role in building trust and enabling digital research with publicly available data (Smallman, 2018). This could take the form of deliberative public engagement events, citizen science projects, as well as more active use of social media and mainstream media outlets to share study results (Entradas et al., 2020; Teng et al., 2019).

Participants across the focus groups varied in their expectations regarding the information they expect to receive about studies when they wish to use their publicly available data for digital research. While consent procedures are part of widely accepted scientific practice for collecting and sharing sensitive data for biomedical research (Bromley et al., 2020; Joly et al., 2015; Wilmes et al., 2023), our study findings suggest that rigorous consent procedures may not be necessary if the publicly available data used in research is perceived as less sensitive. This finding translates to preferences for explicit opt-in consent models for studies with scopes more sensitive to the public or the individuals themselves, such as health and finance status (Spencer et al., 2016). On the other hand, opt-out models would be favorable with broad consent for the general use of less sensitive, anonymized data in digital research with publicly available data. The literature suggests approaches to balance both the requirements of informed consent with the explorative nature of digital research with publicly available data. These include offering broad consent procedures with an information framework to distinguish supported and unsupported scopes of the research study, and implementing dynamic informed consent models through a two-way communication platform between researchers and involved individuals (Christen et al., 2016; Vohland et al., 2021). Also of note, individuals’ perception of the study actors and procedures has shown to influence their willingness to allow researchers to use their data for digital research with publicly available data. This suggests that awareness and understanding of the actors and research methods might suffice to support digital research with publicly available data, eliminating the need for opt-in methods (Aitken et al., 2016).

### Addressing public concerns and implementation challenges around digital research with publicly available data

Participants expressed concern about the level of protection for the publicly available data used in digital research, highlighting uncertainty about whether anonymization ensures adequate data protection. This sentiment is compounded by participants’ limited understanding about how anonymization works, a factor identified in the literature as contributing to concerns about digital research with publicly available data and data sharing in general (Kaye, 2012; Wallace, 2016). In turn, it is not entirely clear which anonymization procedures, ranging from pseudonymization to reducing data to summary-level forms, the public supports for digital research with publicly available data (Haddow et al., 2011). Participants expressed similar concerns about the quality of data collected from public sources and questioned the appropriateness of researchers’ methods in analyzing this data. However, they also noted that their limited understanding of digital research methods hampers their ability to form an informed opinion on supporting or opposing such methods. This notion is reinforced with the hype narrative present in the mainstream media around artificial intelligence, which further challenges the understanding of digital research when artificial intelligence algorithms are used in the methods. Calls for reducing these knowledge gaps are prevalent in the literature (Janssen et al., 2012; Michael and Lupton, 2016; Ruijer et al., 2020), for which adapted approaches of science communication are necessary to empower individuals to make informed decisions about supporting methods in digital research with publicly available data, especially when artificial intelligence methods are involved (Dudo and Besley, 2016; Riesch et al., 2016; Trilles et al., 2020). In general, participants expressed concerns about publicly available data being used for non-research related purposes, particularly when private actors are involved, highlighting the importance of communication strategies to alleviate these concerns (Weingart and Guenther, 2016).

Addressing the concerns voiced by our study’s participants is crucial to establish support in digital research with publicly available data on a national and possibly international scale. However, in our study, we observed that some of the concerns expressed by the participants are based on misconceptions that do not adequately reflect the reality of the procedures, standards and safeguards used in digital research with publicly available data, as well as research in general. This observation raises the question as to whether the same concerns would be present if individuals were adequately informed about the research methods behind digital research with publicly available data and the privacy-security mechanisms taken to address them, such as anonymization. Addressing these concerns in research guidelines and policy should balance participants’ needs while maintaining realistic expectations for their implementation. On the one hand, some of the concerns are addressed through good research practice, which already involves the adoption of appropriate ethical oversight and data protection. On the other hand, additional oversight for research studies, a commonly suggested measure, could be beneficial for guiding digital research with publicly available data, for which similar frameworks in other areas of research have been proposed (Cabili et al., 2021; Vayena and Blasimme, 2018). Nevertheless, researchers should determine the necessity of implementing additional oversight based on the sensitivity and implications of the research study in question. Similarly, when considering adapting approaches for communication, such as through opt-in processes or public communication campaigns, it is important to assess their feasibility and align them with public preferences, preferably through co-design and public engagement approaches (Selin et al., 2017; Woolley et al., 2016). In turn, developing a framework that guides researchers through key considerations of methods and supportive measures to foster their public support would be a valuable next step.

### Ethical considerations and future perspectives

Across the themes, participants discussed their concerns and requirements from an ethical point of view, voicing ethical requirements and normative challenges. The topic of responsibility recurred frequently across the themes and is particularly relevant for an ethical discussion. The topic was discussed on a spectrum between personal responsibility and the responsibility of others. Those emphasizing personal responsibility viewed it as a matter of their agency and responsibility to decide where and how much data they should share. Other participants raised concerns about the possibility of personal responsibility and the ability to sufficiently inform oneself about the risks connected with data use in digital research. These participants saw other actors such as researchers, governmental bodies or ethics committees as having a role of responsibility.

Limits with informed consent such as the opacity of consent and limitations of full understanding are well-known, for example, in medical ethics (Milligan and Jones, 2016; O’Neill, 2003). When regulatory bodies or researchers decide to use consent in digital research with publicly available data, they need to realistically evaluate and determine how such consent can be effective and feasible. Thereby, it is important to consider and evaluate the limits of feasibility and find a balance between public expectations and feasibility requirements. One solution could be to personalize the consent to different participant groups and give them a choice of how they want information to be presented. For example, facilitating the consent presentation with multiple layers and incorporating multimedia tools such as videos, pictures, and text can increase participants’ engagement and empower them to navigate the content at their own pace, fostering a deeper understanding and preventing feelings of being overwhelmed (Antal et al., 2017; De Sutter et al., 2024). Finally, it is necessary to include ethical considerations about a possible systematic exclusion of certain patient groups, such as groups with disabilities that might be automatically excluded when the format is not accessible for them (Shariq et al., 2023).

Similarly, it should be determined how realistic it is to expect that individuals sufficiently inform themselves about data use and bear responsibility in a way that would not be overdemanding and does not become a burden for the person (Heilinger, 2021). This connects to concerns around social justice. More information and education geared to the public might further increase social inequalities (Zajacova and Lawrence, 2018), for example, when the education measures reach mainly people with high levels of literacy. Another factor to be considered is the effectiveness of education and communication measures. There is a well-known privacy paradox when people disclose their personal information despite being worried about their privacy (Bandara et al., 2017; Barth and de Jong, 2017). Hence, it is important to be clear about what informing people exactly means and how communication efforts can be applied in an effective and fair manner.

Discussions in the focus groups also highlighted the importance of addressing emotions as opposed to calculated reasoning when expressing opinions around topics that individuals feel connected to. People often displayed emotions such as fear, enthusiasm, or anger when discussing their concerns and normative expectations, such as for explicit consent procedures. Adressing the emotional factors influencing opinions might be just as important as providing factual information about digital research with publicly available data. Research shows the important role of emotions in decision-making, considering ethical issues (Desmet and Roeser, 2015) and policy (Maor and Capelos, 2023; Pierce, 2021). On the one hand, emotions should be understood as important aspects that reveal personal and moral values that shape individuals’ opinions, concerns and moral considerations (Desmet and Roeser, 2015). Emotions can also influence the scope and direction of public attention (Maor and Capelos, 2023). On the other hand, analyzing emotions could also help with interpreting such individuals’ opinions and moral considerations as they can reveal individual biases underlying those opinions and considerations. Furthermore, there might be a tension between personal moral values and normative considerations detached from specific cases. This friction should be taken into account when evaluating and interpreting individuals’ statements and their relevance for policy making. Hence, analyzing public opinion and reasons should be complemented by taking into account the emotional processes that shape these opinions (Gabehart et al., 2023; Maor and Capelos, 2023).

Finally, discussions in the focus groups revealed that there is a need for clarification of fundamental concepts, such as of privacy, which might currently be undergoing a conceptual shift. Digitalization pertaining to our society has the consequence that many of our concepts are being re-shaped (Löhr, 2023; van de Poel et al., 2023). For example, befriending someone on social media means something different than befriending someone in an offline setting, such as in a classroom. Such a conceptual shift was also reflected in the study. From the discussions, it seemed that participants had a different understanding of private and public information, suggesting a possible shift of the concepts of private and public in the digital space (Keen, 2022; Ravn et al., 2020) compared to the physical space. For some participants, sharing information on social media was considered to be private, for others social media was a public space. It is important to bear these potential conceptual shifts in mind when analyzing public opinions as the meaning of the concept might differ. It might also be fruitful to explicitly discuss such conceptual shifts in future focus groups, which might contribute to more in-depth discussions.

Future communication strategies could take into account emotional factors to increase the public’s awareness and trust in research. This could be achieved by, for example, including emotional content through storytelling, rhetorical tools, such as metaphors, or more engaging communication strategies, such as interacting with data to make the information more relevant for the audience (Lupton, 2018; Taddicken and Reif, 2020). It is also important to ensure that marginalized population groups are not excluded from such communication strategies (Marsh et al., 2023; Okoroji et al., 2023). Further research should also explore how different cultural values and emotional factors influence perceived concerns and requirements by the public. Such research would be particularly informative for formulating targeted communication strategies that can resonate with various audiences. Moreover, such insights hold relevance for international policy formulation, as they contribute to the development of inclusive and culturally sensitive guidelines that could facilitate engagement and adequate oversight in digital research with publicly available data.

## 5. Strengths and limitations

This study presents a first nationwide assessment of a broad spectrum of age groups, political affiliations and cultural regions in Switzerland that assesses public perspectives on digital research with publicly available data. The findings from this study are particularly informative for shaping guidelines on the conduct of digital research with publicly available data in Switzerland and, ultimately, to inform policymaking. Nevertheless, the study has limitations. First, there could be an overlap between the identified themes, as the focus groups were not entirely structured to avoid influencing public opinion. This might lead to different interpretations of the themes and their respective categories. Second, cultural comparisons between the focus groups from the different language regions were not possible given the lack of explicit measures available to make direct conclusions on the observed differences, which limits the ability to directly inform a guideline tailored to different cultural contexts in Switzerland. Finally, the study is limited in addressing the broader cultural and political context beyond Switzerland, particularly in regions affected by conflicts, where data availability may pose security risks which deserve more attention in future research.

## 6. Conclusion

Our findings emphasize public support for digital research with publicly available data, contingent on meeting specific requirements. To legitimize digital research with publicly available data, it is essential to foster public trust by actively involving the public from the outset to meaningfully guide research efforts and empower them to decide how their data should be used. Equally important is the sustained engagement with the public by clearly communicating the societal impact achieved through the use of their data and how their concerns are being addressed. Concerns about data use should be addressed beyond study objectives, especially if data is collected on more sensitive topics, by highlighting efforts taken around privacy preservation, anonymization, reliable research methods, and possible external oversight. The development of a guiding framework for researchers to inform the publicly supported use of publicly available data in digital research is identified as a valuable next step. Future research should explore emotional aspects of awareness and trust, considering cultural differences and customizing public communication strategies accordingly.

## Supplemental Material

sj-docx-1-pus-10.1177_09636625251330575 – Supplemental material for Public views on research with publicly available data in Switzerland: Implications for digital research, science communication, and policy Supplemental material, sj-docx-1-pus-10.1177_09636625251330575 for Public views on research with publicly available data in Switzerland: Implications for digital research, science communication, and policy by Paola Daniore, Jana Sedlakova, Federica Zavattaro, Zoé Huber, Melanie Knieps, Manon Haulotte, Togbé Agbessi Alangue, Artemis Faulk, Viktor von Wyl, Yaniv Benhamou and Felix Gille in Public Understanding of Science
