# Supplementary material for: Public views on research with publicly available data in Switzerland: Implications for digital research, science communication, and policy
Source: Public Underst Sci. 2025 May 22;34(8):988–1008. doi: 10.1177/09636625251330575 (PMC12535623; doi:10.1177/09636625251330575)
Supplement: sj-docx-1-pus-10.1177_09636625251330575 – Supplemental material for Public views on research with publicly available data in Switzerland: Implications for digital research, science communication, and policy [file sj-docx-1-pus-10.1177_09636625251330575.docx]

**Supplemental material for article “Public views on research with publicly available data in Switzerland: Implications for research, science communication, and policy”**

Paola Daniore, PhD, Jana Sedlakova, PhD, Federica Zavattaro, MSc, Zoé Huber, MLaw, Melanie Knieps, PhD, Manon Haulotte, BA, Togbé Agbessi Alangue, MLaw^4^ Artemis Faulk^,^ BA, Viktor von Wyl, PhD, Yaniv Benhamou, PhD, Felix Gille, Ph

Supplemental Material: Table of Contents

[Supplemental Data 1 2](#_Toc192690979)

[Case studies presented and structure of the focus group 2](#_Toc192690980)

[Supplemental Data 2 3](#_Toc192690981)

[Examples of MaxQDA coded segments 3](#_Toc192690982)

[Supplemental Table 1 8](#_Toc192690983)

[Subthemes identified in first analysis round 8](#_Toc192690984)

# **Supplemental Data 1**

## **Case studies presented and structure of the focus group**

Case studies presented

**Case study 1**: Data Sharing For Precision Medicine: Policy Lessons And Future Directions (doi: 10.1377/hlthaff.2017.1558)

**Case study 2**: Assessing Public Opinion on CRISPR-Cas9: Combining Crowdsourcing and Deep Learning (doi: 10.2196/17830)

**Case study 3**: Natural Language Processing of Social Media as Screening for Suicide Risk (doi: 10.1177/1178222618792860)

**Case study 4**: Using text mining and sentiment analysis to analyse YouTube Italian videos concerning vaccination (doi: 10.1186/s12889-020-8342-4)

Structure of the focus groups

We conducted a total of 10 focus groups. The first four included individuals from the German-language speaking region of Switzerland. Among these, one focus group was conducted in person and the remaining three were conducted online. The participants of these focus groups discussed case studies 1 and 2. The second four focus groups included individuals from the French-language speaking region of Switzerland. All of these focus groups were conducted online. The participants of these focus groups discussed case 3. The last two focus groups included individuals from the Italian-language speaking region of Switzerland. All of these focus groups were conducted online. The participants of these focus groups discussed case 4.

# **Supplemental Data 2**

## **Examples of MaxQDA coded segments**

If social media data can be exploited by anyone, everyone needs to be aware and teach people not to be slaves and measure the risks.

Code: ● GO_PERCEPTION_Subtheme > GO_Public awareness Weight score: 0

20230705_French_Group_2, Column: 1 | Row: 1

If social media data can be exploited by anyone, everyone needs to be aware and teach people not to be slaves and measure the risks.

Code: ● Goal_TRANSPARENCY_Subtheme > Goal_Public education Weight score: 0

20230705_French_Group_2, Column: 1 | Row: 1

I don't mind giving a machine the power to say that this or that person has suicidal tendencies.

Code: ● GO_DATA_SENSITIVITY_Subtheme > GO_Indifference towards DM Weight score: 0

20230705_French_Group_2, Column: 3 | Row: 1

No, I don't agree to share my data to prevent suicide. It has to be voluntary (opt-in)

Code: ● PM_OPT-IN/OPT-OUT_Subtheme > PM_Opt-in / Opt-out Weight score: 0

20230705_French_Group_2, Column: 5 | Row: 1

It's better if it's for a good purpose than a commercial one.

Code: ● GO_INCENTIVES_Subtheme > GO_Public benefit Weight score: 0

20230705_French_Group_2, Column: 7 | Row: 1

It's better if it's for a good purpose than a commercial one.
 For commercial purposes, our data should definitely not be used

Code: ● A_MOTIVATIONS_Subtheme > A_Use of data depends on actor and intent Weight score: 0

20230705_French_Group_2, Column: 7 | Row: 1

For commercial purposes, our data should definitely not be used

Code: ● A_CONCERN_Subtheme > A_Monetary intentions of private companies problematic Weight score: 0

20230705_French_Group_2, Column: 7 | Row: 1

This is the price of the gratuity that we pay. Give our data in exchange

Code: ● GO_ACCOUNTABILITY_Subtheme > GO_Self responsibility Weight score: 0

20230705_French_Group_2, Column: 9 | Row: 1

Once the research community has used data, it must define the framework for the use of the data, determine who has access to it and how the data is destroyed.

Code: ● DC_GOVERNANCE_Theme > DC_Governance framework for data use, access and timeline Weight score: 0

20230705_French_Group_2, Column: 11 | Row: 1

I trust the government more to collect and use data for public utility

Code: ● A_ENJOY_TRUST_Subtheme > A_Trust in public institutions Weight score: 0

20230705_French_Group_2, Column: 13 | Row: 1

Universities that publish articles should check whether researchers have asked people for consent before the study

Code: ● A_TRANSPARENCY_Subtheme > A_Informed consent Weight score: 0

20230705_French_Group_2, Column: 15 | Row: 1

Consumer associations can also be proactive in communicating information on data collection

Code: ● A_GOVERNANCE_Theme > A_Consumer associations Weight score: 0

20230705_French_Group_2, Column: 17 | Row: 1

Switzerland's fines are too low for large companies that break the law

Code: ● PM_LEGISLATION_Subtheme > PM_Regulation of private sector Weight score: 0

20230705_French_Group_2, Column: 19 | Row: 1

Everyone needs to read the General Terms of Use (GTU) carefully.

Code: ● GO_T&Cs_Subtheme > GO_T&Cs are not read Weight score: 0

20230705_French_Group_2, Column: 1 | Row: 2

There's a hotline (call center) but you have to call it and not everyone does. So, as far as the study is concerned, the aim of trying to prevent suicide is laudable enough, but it's no guarantee of privacy.

Code: ● DC_CONCERN_Subtheme > DC_Privacy Weight score: 0

20230705_French_Group_2, Column: 3 | Row: 2

Giving explicit consent to use data should be the norm. No one reads these terms and conditions of use, they are too long

Code: ● GO_T&Cs_Subtheme > GO_Preference to refuse T&Cs by default (Opt-in) Weight score: 0

20230705_French_Group_2, Column: 5 | Row: 2

Insurance companies should not make combinations with the data collected, this is not correct

Code: ● DA_DATA_RE-USE_Subheme > DA_Ambivalent acceptance of data re-use Weight score: 0

20230705_French_Group_2, Column: 7 | Row: 2

I don't like it, but I'm convinced that my data is being reused

Code: ● DA_DATA_RE-USE_Subheme > DA_Ambivalent acceptance of data re-use Weight score: 0

20230705_French_Group_2, Column: 9 | Row: 2

There must be a guarantee that data will not be shared or resold

Code: ● GO_TRANSPARENCY_Subtheme > GO_Guarantee data not shared or re-sold Weight score: 0

20230705_French_Group_2, Column: 11 | Row: 2

If the data are collected by governments with totalitarian tendencies, dysfunctional institutions, I will be reluctant to give my consent. But if I am sure that the legal framework is clear, I will give my consent, but with a clear idea of the purpose of the collection.

Code: ● Goal_TRANSPARENCY_Subtheme > Goal_Informed consent Weight score: 0

20230705_French_Group_2, Column: 13 | Row: 2

If the data are collected by governments with totalitarian tendencies, dysfunctional institutions, I will be reluctant to give my consent. But if I am sure that the legal framework is clear, I will give my consent, but with a clear idea of the purpose of the collection.

Code: ● PM_PRECAUTIONS_Subtheme > PM_Define meaning and purpose Weight score: 0

20230705_French_Group_2, Column: 13 | Row: 2

But if I am sure that the legal framework is clear

Code: ● PM_FRAMEWORK_Subtheme > PM_Legislative framework Weight score: 0

20230705_French_Group_2, Column: 13 | Row: 2

Researchers need to popularize their research and hold debates on TV and other public information channels.

Code: ● PM_COMMUNICATION_EVENT_Subtheme > PM_Organize public debates Weight score: 0

20230705_French_Group_2, Column: 15 | Row: 2

Establish an authorisation system, e.g. a data protection officer who sets the framework within which data will be used

Code: ● PM_FRAMEWORK_Subtheme > PM_Data protection framework Weight score: 0

20230705_French_Group_2, Column: 17 | Row: 2

No one reads these terms and conditions of use, they are too long

Code: ● GO_T&Cs_Subtheme > GO_T&Cs are not read Weight score: 0

20230705_French_Group_2, Column: 1 | Row: 3

No, I don't agree to share my data to prevent suicide. It has to be voluntary (opt-in)

Code: ● PM_OPT-IN/OPT-OUT_Subtheme > PM_Opt-in / Opt-out Weight score: 0

20230705_French_Group_2, Column: 3 | Row: 3

I am not in favour of reusing my data for purposes other than those for which I have consented

Code: ● DA_DATA_RE-USE_Subheme > DA_Ambivalent acceptance of data re-use Weight score: 0

20230705_French_Group_2, Column: 5 | Row: 3

You need to have a clear idea of who is collecting what and for what purpose, and then you're free to decide, but first you need to know

Code: ● A_TRANSPARENCY_Subtheme > A_Transparency Weight score: 0

20230705_French_Group_2, Column: 7 | Row: 3

You need to have a clear idea of who is collecting what and for what purpose, and then you're free to decide, but first you need to know

Code: ● DC_TRANSPARENCY_Subtheme > DC_Transparency Weight score: 0

20230705_French_Group_2, Column: 7 | Row: 3

It's the researcher's responsibility to ensure transparency, with institutional supervision.

Code: ● PM_RESPONSIBILITY_Subtheme > PM_Transparency responsibility of researcher Weight score: 0

20230705_French_Group_2, Column: 11 | Row: 3

Regulation is needed at state level, but it should be global, because data crosses borders.

Code: ● PM_LEGISLATION_Subtheme > PM_Regulatory function of state Weight score: 0

20230705_French_Group_2, Column: 13 | Row: 3

It's not right to be invaded by advertising that comes from the analysis of our data.

Code: ● PM_COMMUNICATION_SOURCES_Subtheme > PM_Limit advertising from data analysis Weight score: 0

20230705_French_Group_2, Column: 15 | Row: 3

If data are secured and anonymized, I agree, but I think it would be very difficult to do in the case of suicide.

Code: ● GO_DATA_PRECAUTIONS_Subtheme > GO_Data anonymity Weight score: 0

20230705_French_Group_2, Column: 3 | Row: 4

They analyze data and then give a message to our loved ones, it goes too far

Code: ● UoR_CONCERN_Subtheme > UoR_Data used against them later on Weight score: 0

20230705_French_Group_2, Column: 5 | Row: 4

Isn't research biased if we only use the data of people who have given their consent? AI does a preliminary sorting that skews the search.

Code: ● A_DATA_QUALITY_Subtheme > A_Data validity Weight score: 0

20230705_French_Group_2, Column: 7 | Row: 4

There are issues related to data hacking. We are not immune to slippages

Code: ● DC_DATA_QUALITY_Subtheme > DC_Validity of data Weight score: 0

20230705_French_Group_2, Column: 15 | Row: 4

It is less problematic if it is the public authorities that collect and analyze the data, they are not supposed to have dubious interests.

Code: ● A_ENJOY_TRUST_Subtheme > A_Trust in public institutions Weight score: 0

20230705_French_Group_2, Column: 7 | Row: 5

# **Supplemental Table 1**

## **Subthemes identified in first analysis round**

| **Theme** | **Subthemes** |
| --- | --- |
| *Awareness of involvement in digital research with publicly available data* | - Perception - Implications - Transparency - Terms and conditions |
| *Communication strategies to increase engagement in research* | - Communication event - Communication sources - Direct communication - Public communication |
| *Individual and public expectations with digital research with publicly available data* | - Individual accountability - Individual benefit - Individual concern - Individual incentives - Individual requirements - Source of contact - Public benefit |
| *Requirements for the appropriate use of public data in digital research with publicly available data* | - Approved data types - Data limits - Data precautions - Data protection - Data quality - Data sensitivity - Data re-use - Motivation of data choice - Not approved data types |
| *Requirements for the appropriate use of digital research with publicly available data methods with public data* | - Approved digital research with publicly available data methods - Concerns with digital research with publicly available data methods - Precautions with digital research with publicly available data methods - Quality of digital research with publicly available data methods - Requirements from digital research with publicly available data methods - Support of digital research with publicly available data methods |
| *Regulatory precautions for future digital research with publicly available data* | - Advisory - Ethics - Framework - Governance - Legislation - Precautions - Opt-in/Opt-out - Oversight - Regulatory requirements - Responsibility |
| *Sources of (mis)trust with digital research with publicly available data* | - Ambivalent trust - Approved data - Concerns - Data quality - Enjoy trust - Governance - Individual accountability - Lack of trust - Not approved data - Transparency |
